# Supplementary material for: Causal interplay between lactose intolerance and gut microbiota: a combined bidirectional Mendelian randomization and in vivo validation study
Source: Front Nutr. 2026 Jun 1;13:1803337. doi: 10.3389/fnut.2026.1803337 (PMC13265576; doi:10.3389/fnut.2026.1803337)
Supplement: Supplementary file 1 [file Data_Sheet_1.zip › supplementary materials/Forward/leave one out cross validation/ebi-a-GCST90027623.finngen_R12_E4_LACTONAS.pdf]

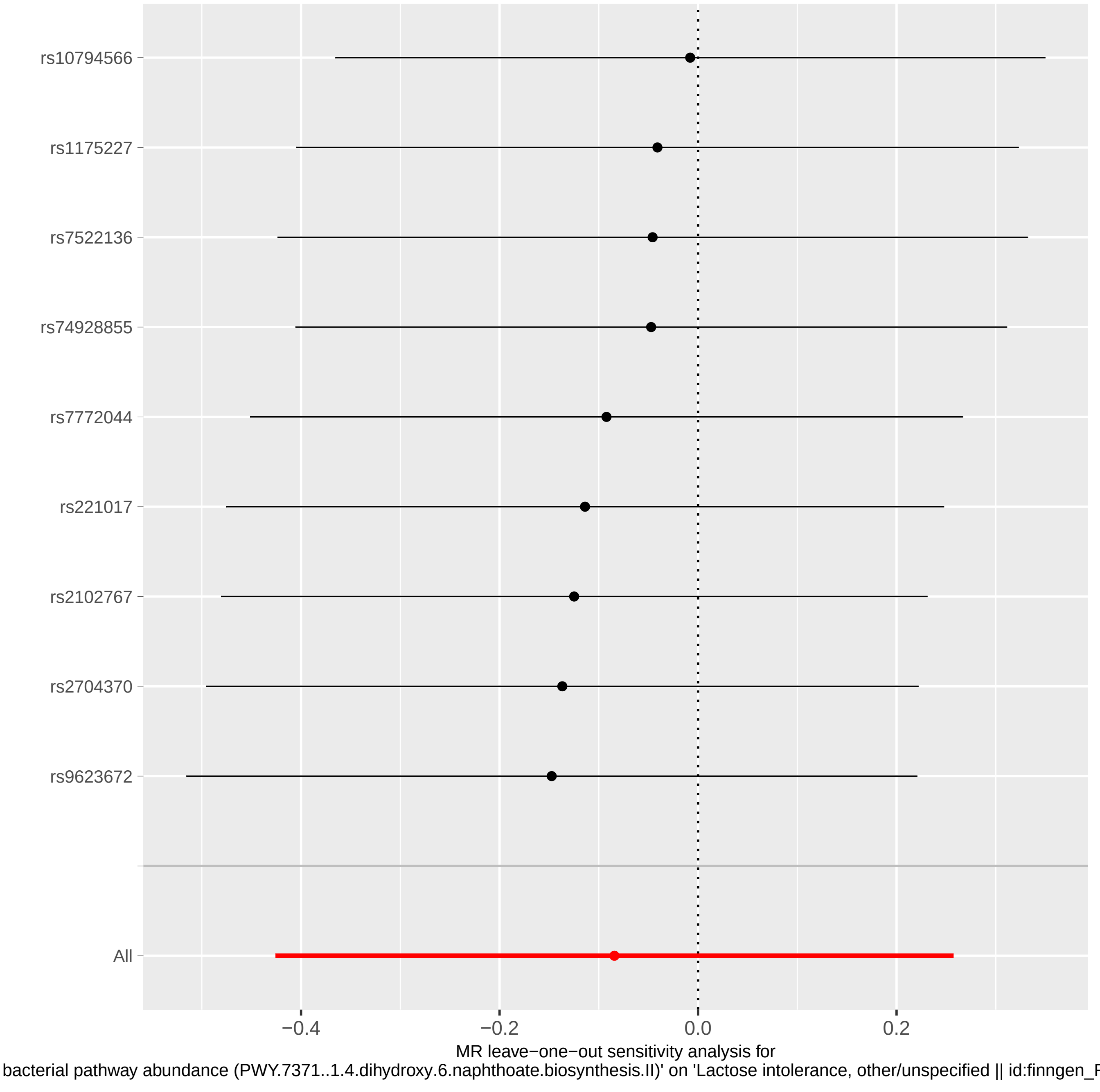

rs10794566
rs1175227
rs7522136
rs74928855
rs7772044
rs221017
rs2102767
rs2704370
rs9623672
All
−0.4
−0.2
0.0
0.2
MR leave−one−out sensitivity analysis for
bacterial pathway abundance (PWY.7371..1.4.dihydroxy.6.naphthoate.biosynthesis.II)' on 'Lactose intolerance, other/unspecified || id:finngen_F
